# The role of mind body interventions in the treatment of irritable bowel syndrome and fibromyalgia

**DOI:** 10.3389/fpsyt.2022.1076763

**Published:** 2022-12-22

**Authors:** Zarmina Islam, Adrijana D’Silva, Maitreyi Raman, Yasmin Nasser

**Affiliations:** ^1^Department of Community Health Sciences, Cumming School of Medicine, University of Calgary, Calgary, AB, Canada; ^2^Division of Gastroenterology and Hepatology, Department of Medicine, University of Calgary, Calgary, AB, Canada; ^3^Snyder Institute for Chronic Diseases, Cumming School of Medicine, University of Calgary, Calgary, AB, Canada

**Keywords:** IBS–irritable bowel syndrome, fibromyalgia (FM), mind-body interventions, yoga, cognitive behavioral therapy (CBT)

## Abstract

**Introduction:**

Irritable bowel syndrome and fibromyalgia share similar pathophysiologic mechanisms including sensitization of peripheral and central pain pathways, autonomic dysfunction and are often co-diagnosed. Co-diagnosed patients experience increased symptom severity, mental health comorbidities, and decreased quality of life. The role of mind-body interventions, which have significant effects on central pain syndromes and autonomic dysregulation, have not been well-described in co-diagnosed patients. The aim of this state-of-the art narrative review is to explore the relationship between irritable bowel syndrome and fibromyalgia, and to evaluate the current evidence and mechanism of action of mind-body therapies in these two conditions.

**Methods:**

The PubMed database was searched without date restrictions for articles published in English using the following keywords: fibromyalgia, irritable bowel syndrome, mind-body interventions, cognitive behavioral therapy, mindfulness based stress reduction, and yoga.

**Results:**

Mind-body interventions resulted in improved patient-reported outcomes, and are effective for irritable bowel syndrome and fibromyalgia individually. Specifically, cognitive behavioral therapy and yoga trials showed decreased symptom severity, improved mental health, sleep and quality of life for both conditions individually, while yoga trials demonstrated similar benefits with improvements in both physical outcomes (gastrointestinal symptoms, pain/tenderness scores, insomnia, and physical functioning), mental health outcomes (anxiety, depression, gastrointestinal-specific anxiety, and catastrophizing), and quality of life, possibly due to alterations in autonomic activity.

**Conclusion:**

Mind-body interventions especially CBT and yoga improve patient-reported outcomes in both irritable bowel syndrome and fibromyalgia individually. However, limited available data in co-diagnosed patients warrant high quality trials to better tailor programs to patient needs.

## 1 Introduction

Irritable Bowel Syndrome (IBS) is a prevalent disorder that affects 7–21% of the population worldwide, and 12% of Canadians (1, 2). IBS is characterized by abdominal pain and altered bowel habits and is classified according to the primary bowel habit: IBS-D (diarrhea predominant), IBS-C (constipation predominant), and IBS-M (mixed), with some patients migrating between subtypes (1, 2). The etiology of IBS is multifactorial with aberrant brain-gut interactions (1) at its core. Patients with IBS have a poor quality of life owing to the severity of gut symptoms as well as associated comorbidities, including somatic pain disorders and psychiatric disorders (1). High symptom burden in IBS is associated with lost productivity and work absenteeism, accounting for at least $20 billion a year and cost of $9,993 per patient, and 3.5 million physician visits in the United States (3–5).

Current literature suggests that a strong relationship exists between fibromyalgia (FM) and IBS (6). FM is characterized by chronic widespread pain, headaches, sleep disturbances, difficulty concentrating, depression, and fatigue (7, 8). FM has a global prevalence of 2.7% [range 2–8% (9)]. Like IBS [3:1 ratio (4)], FM is more prevalent in women compared to men [6:1 (9)]. FM costs $8,561 per patient per year in lost productivity and work absenteeism, with direct medical costs that are three times higher than in patients without FM, highlighting its significant burden (10, 11).

FM and IBS have substantial symptom overlap and are frequently co-diagnosed (6). They have common comorbidities including other functional gastrointestinal disorders, pain syndromes (12) and psychiatric conditions including depression (13), suggesting that they share a common pathogenesis. Both disorders are difficult to treat with conventional pharmacotherapies (14–16). Up to 50% of IBS patients and 91% of FM patients seek non-pharmacologic or complementary and alternative treatments to manage their symptoms (17, 18). Thus it is critical to understand how evidence-based non-pharmacologic therapies can be used to treat these disorders.

Mind-body interventions (MBI) are effective in symptom improvement, stress relief, cognitive flexibility, and improved attention and concentration, suggesting these may modify central pain pathways, and/or autonomic dysfunction in both IBS and FM (3, 19–22). It is imperative to understand how MBIs can be used as adjunctive treatments in co-diagnosed IBS and FM. The aim of this study is to review the current literature to describe the prevalence, comorbidities, and shared pathophysiology of co-diagnosed IBS and FM. We also discuss the rationale and evidence for MBI as a therapeutic strategy in these disorders. We focus on mindfulness, cognitive behavioral therapy and yoga because of their popularity among patients and the quality of available clinical studies.

## 2 Methodology

Our methodology consisted of a PubMed search without date restrictions for articles published in English using the following keywords: Fibromyalgia, Irritable Bowel Syndrome, Mind-body interventions, Cognitive behavioral therapy (CBT), Mindfulness based stress reduction (MBSR), and Yoga. Variations of these keywords were also used; mindfulness, MBSR, MBI, IBS, and FM/Fibromyalgia Syndrome (FMS). Both primary and secondary articles were used to synthesize this review.

## 3 The relationship between IBS and FM

IBS patients report symptoms of bloating, abdominal pain, and altered bowel habits such as constipation or diarrhea (1). Extraintestinal symptoms include headache, insomnia, fatigue, and palpitations (4). FM presents with unexplained musculoskeletal and widespread pain along with fatigue, sleep disturbances, and altered bowel habits (4, 6, 12). Diagnostic criteria involves assessment of defined tender points using the 2016 fibromyalgia survey with widespread pain on both sides of the body (23), although variability exists in presentations (12).

A systematic review (*n* = 14 studies) reported the prevalence of IBS in FM to be 32.5% (range 28–59%), whereas 73% of patients with FM reported altered bowel habits (6). Despite shared comorbidities and symptoms, the prevalence of FM in IBS has not been well-defined. There is a discordance in prevalence estimates, ranging from 48% (range 32–77%) to 12.9% (95% CI 12.7–13.1) from a systematic review (*n* = 30 studies) and meta-analysis (*n* = 65 studies), likely as a consequence of differing study designs (24, 25).

Amongst FM patients, bowel symptoms occur frequently: bloating (65.4%), abdominal pain (57.1%), fecal incontinence (56%), constipation (52.9%), alternating diarrhea and constipation (21.3%), and diarrhea alone [6% (6)]. Interestingly, FM predominates in patients with IBS-C (6). Both FM and IBS affect women more and overlap with depression, anxiety, sleep difficulties, fatigue, and chronic headaches (1, 6, 12). Psychiatric disorders are highly prevalent in both conditions. For instance, 30–50% and 30% of patients with functional gastrointestinal disorders report anxiety, and depression, respectively (26). In IBS, a prevalence of 39.1 and 23.8% exists for anxiety and depression, respectively, affecting the IBS-C type most (27, 28). Moreover, 38% of IBS patients report suicidal ideation (29). In comparison, FM has a prevalence of 32% for mood disorders, 63% for depression, with 32.5% of patients reporting suicidal ideation (13, 24, 30).

## 4 Common pathophysiologic basis in IBS and FM

### 4.1 Central sensitization and altered neurotransmission

An altered central pain state, characterized by increased neuronal excitability resulting in hyperalgesia (increased pain intensity from a painful stimulus), as well as allodynia (pain caused by a non-painful stimulus), is the first proposed common mechanism underpinning FM and IBS (31). Both FM and IBS patients show enhanced activation of ascending excitatory pain pathways, and dampening of descending inhibitory pain pathways (12, 32, 33). This results in heightened activation of central pain circuits and in the processing of negative emotions in the brain. Patients with IBS and FM individually show greater activation of brain areas associated with pain, negative emotions, memory retrieval, and attention to sensory stimuli compared to healthy participants (26, 34–37). In FM, functional MRI studies demonstrate heightened pain processing in subcortical and cortical regions in response to mild pressure that is perceived as normal touch for those without FM (38). In IBS, MRI studies demonstrate abnormal brain responses to painful visceral stimuli, such as rectal distention (39) as well as abnormal brain activity and connectivity at rest (26, 40) suggesting that abnormal central pain processing is a key component of both IBS and FM.

### 4.2 Somatic/visceral hypersensitivity

IBS is characterized by visceral hypersensitivity while FM is characterized by somatic hypersensitivity. Those with co-diagnosed IBS and FM show somatic hyperalgesia with lower pain thresholds and higher pain frequency and severity, whilst those with only IBS demonstrate somatic hypoalgesia (41).

Peripheral sensitization of nociceptors (pain-sensing neurons) contributes to hypersensitivity in both IBS (35) and FM (7). Peripheral nociceptors, either at the level of the gut wall, or at the level of the skin and joints, express receptors for mediators (e.g., proteases, cytokines, histamine, and bradykinin) which are released in response to cell damage or injury. These mediators can sensitize nociceptors, leading to increased neuronal excitability. In turn, nociceptors release substance P and calcitonin gene related peptide, which augment the inflammatory response at the level of the periphery and activate central pain pathways, thus contributing to central sensitization (7, 35).

### 4.3 Autonomic dysfunction

Both IBS and FM are associated with increased sympathetic tone and activation of the hypothalamic-pituitary-adrenal (HPA) axis, which is associated with disturbances in gut motility (6, 24, 42, 43). This suggests why MBIs may be effective for both disorders as they are thought to increase parasympathetic activity (3) and dampen sympathetic outflow.

### 4.4 Gut microbial dysbiosis

An altered gut microbiome is hypothesized to contribute to the pathophysiology of IBS and FM, although is more extensively characterized in IBS. Dysbiosis, or a change in the gut microbiome composition, has been shown in both disorders (44–46), with an altered Firmicutes to Bacteroidetes ratio observed at a phyla level, although the data are heterogeneous. IBS is associated with a high Firmicutes to Bacteroidetes ratio (45, 46) whereas in a study comparing 54 FM patients and 36 healthy individuals, a low Firmicutes to Bacteroidetes ratio (44) was observed. A decrease in Firmicutes has also been associated with major depressive disorder which is comorbid in IBS and FM (47). However, it is unknown whether these changes in the gut microbiome are a cause or consequence of altered gut motility. Further studies are warranted to understand the causative role of dysbiosis in both conditions.

### 4.5 Psychological basis

There is strong evidence that psychological comorbidities in IBS increase stress reactivity and amplify somatic sensations (24). Patients with IBS or FM report increased adverse early life events (48), a perceived lack of social support, and increased association of stressful life events to symptoms (26, 49). In addition, IBS and FM share a behavioral component called “catastrophization” (envisioning the worst possible scenario for an action or exaggerating a painful experience) which correlates with pain severity, presenting a potential therapeutic target for MBIs (1, 24).

## 5 Impact on quality of life

### 5.1 Psychological

A meta-analysis found a strong correlation between medically unexplained symptoms and increased depression/anxiety in IBS and FM (50). In IBS, a positive correlation was seen between somatic and psychiatric comorbidities, increased health care seeking, and reduced quality of life (51). Major depressive disorder is the most common psychiatric comorbidity in FM and IBS (4, 13). However, FM is characterized by lower anxiety scores than IBS (50).

### 5.2 Sleep and fatigue

Sleep disturbances contribute to pain, as lack of sleep impairs descending pain inhibitory pathways, impairing an individual’s ability to cope with pain (52). Sleep disorders are highly common in IBS and FM, with studies estimating a prevalence of 33% in IBS (48) and 92.9% in FM (53). More than 50% of FM patients meet criteria for insomnia; non-restorative sleep in these patients is associated with heightened pain, cognitive arousal and catastrophization (32). FM patients report morning stiffness, fatigue, and pain; hence improving the sleep quality by employing exercise is effective (54). In addition, fatigue contributes to poor health in both conditions. There is a median comorbidity of 51% for chronic fatigue syndrome in IBS and 76% in FM. Patients with comorbid chronic fatigue have 57% loss of productivity and 37% decline in household income (48, 55, 56) compared to those without. Furthermore, patients with co-diagnosed FM and IBS experience increased fatigue and symptom severity compared to those with FM or IBS alone (6). Taken together, a co-diagnosis of both FM and IBS results in significantly increased fatigue, poor sleep, and impaired quality of life, suggesting a need for therapies aimed at improving these common symptoms. Given the role of stress and anxiety in exacerbating chronic pain in both conditions, it would be important to engage patients in therapies which address these concerns.

## 6 Mind-body interventions

Mind-body interventions (MBI) are alternative treatment options that allow active participation of patients in their health. This is done through introspective practices that involve self-observation, meditation, relaxation exercises such as breathing, and non-judgmental acceptance of both internal (emotions, breathing, etc.) and external events (noises, smells, etc.) known as mindfulness (57). This review will focus on: (a) Mindfulness MBIs such as Mindfulness-based stress reduction, Mindfulness-Based Cognitive Therapy, Mindful Socioemotional Regulation Intervention, and Tai Chi; (b) Cognitive Behavioral Therapy (CBT); and (c) Yoga. A summary of randomized controlled trials examining MBIs for FM and IBS is found in Tables 1, 2, respectively.

**TABLE 1:** Mind-body randomized control trials for Fibromyalgia.

Study	Intervention and population	Comparison	Inclusion criteria	Assessments	Results (primary outcomes)	Results (secondary outcomes)	Attrition	Compliance	Adverse events
Carson et al. (59, 60) (United States)	Intervention: 8-week yoga of awareness program Population: Diagnosed FM female patients—ACR criteria Mean age: 51.4 (SD: 13.7) (*n* = 25) Components: Yoga of Awareness program: Gentle stretching, mindfulness meditation, breathing techniques, presentations on yoga application to coping, and group discussions. Duration and frequency: 8-week program once-per-week 120 min group classes (7–12 patients in each group) Home practice: Encouraged home practice for 20–40 min per day, 5–7 days per week guided by a DVD	Mean age: 55.8 (8.9) (*n* = 28) wait-listed standard care	53 female FM patients (≥21 years), ACR criteria for at least 1 year, treatment for FM ≥ 3 months	Baseline (2 weeks before yoga intervention), post-treatment	Patients in the yoga group showed an Improvement in pain, fatigue, and mood components. At 3-month follow-up, myalgic score and number of tender points was reduced.	Not reported	Not reported	Yoga completion rate: 91%	Not reported
Da Silva et al. (96) (Brazil)	Intervention: 8-week relaxing yoga Population: Diagnosed FM female patients—ACR criteria Mean age: 46.3 ± 8.9 (*n* = 17) Components: Relaxing yoga (RY): Simple postures of stretching according to Gharote’s methodology, diaphragmatic yogic breathing, relaxation technique focusing on attention to major body parts, principles of yogic philosophy were read by the therapist at the end Duration and frequency: 8 weekly sessions of relaxing yoga (RY) 50 min each Home practice: Encouraged to maintain regular yoga practice	Mean age: 44.4 ± 11.0 (*n* = 16) relaxing yoga plus touch (RYT) with Tui Na Tui Na comprised of sliding (“tui fa”) and pressuring (“na fa”) maneuvers. Home practice: Not reported	40 FMS women (25–60) ACR 1990 criteria	Baseline (1 week before start of treatment), 4–6 weeks post-treatment	RY and RYT showed decreased pain scores with RY pain scores much lower in follow-up. Addition of touch contributed to greater improvement whereas yoga reduced pain.	Not reported	Not reported	Not reported	Not reported
Schmidt et al. (61) (Germany)	Intervention: 8 week MBSR plus yoga Population: Diagnosed FM female patients—ACR criteria Mean age: 53.4 ± 8.7 (*n* = 53) Components: MBSR including Yoga: Mindfulness practices, yoga postures, and mindfulness during stressful situations, and social interactions. Duration and frequency: 2.5-h session every week for 8 weeks and an additional 7-h all day session on a weekend. Groups of up to 12 participants and one instructor were set up. Home practice: Daily homework assignments of 45–60 min.	Active control intervention Mean age: 51.9 ± 9.2 (*n* = 56) Wait-list control group Mean age: 52.3 ± 10.9 (*n* = 59) Study used both active and wait-list control. Active control group was similar to MBSR intervention group, however active control did not have a 7-h all day session. Components include Jacobson Progressive muscle Relaxation training (PMR), and fibromyalgia-specific gentle stretching exercises.	Women with FM (18–70 years), ACR criteria, German language, interest in participating	Short-term follow-up, 8 weeks postintervention	All groups showed an improvement in HRQoL at short-term follow-up, whereas MBSR group showed more benefits pre to post-treatment.	Positive change in 6 of 8 outcomes for MBSR. Active treatment group at postintervention showed decrease in anxiety compared to waitlist and MBSR show higher mindfulness compared to active group.	Not reported	Not reported	Not reported
Rudrud (97) (United States)	Intervention: 8 week gentle Hatha yoga Population: physician diagnosed FM female patients Mean age: not reported (*n* = 10) Components: Nostril breathing, gentle standing poses, seated postures, and body awareness Duration and frequency: 2 times per week for 8 weeks Home practice: Not suggested to participants, but reported in recommendations	None	Women with physician diagnosed FM aged 39–64 years were included in this study. Participants were required to have no other health conditions that would limit their ability to participate in yoga.	Baseline, post-intervention	Quantitative: 70% participants report decrease in FM related pain. Tender point evaluation also indicated reduced pain. Qualitative: Participants report experiencing more pain in the first few weeks of classes.	Not reported	2/10 withdrew	2/10 did not complete the program	Not reported
Lazaridou et al. (76) (United States)	Intervention: 4 week CBT Population: Diagnosed FM female patients—ACR criteria Mean age: 45.7 ± 12.2 (*n* = 8) Components: CBT: Emotional regulation, pain self-management, catastrophizing, relaxation techniques, etc. Duration and frequency: CBT for 4 weeks. 60–to 70-min visits each week Home practice: Written exercises	(*n* = 8) Fibromyalgia education (control): received CBT following completion of their posttreatment No homework.	16 FM patients. 18 or older, rheumatologist-diagnosed FM for at least 1 year, Wolfe et al. ACR criteria, PCS score of at least 21.	Baseline, post-treatment, and 6-month follow-up.	Improvement in PCS and BDI pain interference scores at 6-month follow-up.	Brain connectivity analysis shows reductions in PCS associated with alterations in S1 connectivity.	1 participant dropped out	Not reported	Not reported
Lazaridou et al. (98) (United States)	Intervention: 6 week yoga Population: Diagnosed FM patients—ACR 2011 criteria Mean age: 48.5 ± 13.9 (*N* = 42) Components: Yoga: asanas, pranayama, pratyahara, and meditation. Duration and frequency: Yoga for 6 weeks, 1.5 h sessions in groups of roughly 10 participants. Home practice: 30 min videos for regular home practice	No control comparison group	18–75 years of age with a diagnosis of FM according to 2011 criteria for over 6 months, average pain score ≥4/10, sleep disturbance defined as Pittsburgh Sleep Quality Index (PSQI) score ≥ 5, speaks English, access to technology, and physically able to commit to yoga	Baseline, 6 weeks	Improvement in pain symptoms. Greater home practice, yielded greater decrease in pain Significant association between anxiety and catastrophizing	Not reported	10 participants dropped out	74% (*N* = 36) completion rate	Not reported
Wang et al. (62) (United States)	Intervention: 12–24 weeks Tai Chi Population: Diagnosed FM patients—ACR 1990 and 2010 criteria Mean age: 1 session × 12-week group: 53.0 (SD: 12.6); 2 session × 12 week group: 52.1 (10.3); 1 session × 24 week group: 50.8 (11.8); 2 session × 24 week group: 52.1 (13.3) (*n* = 151) Components: Tai Chi: Warm-up, tai chi principles, meditative exercises, breathing techniques, and relaxation. Duration and frequency: One of four classic tai chi interventions, 60 min each, 12 or 24 weeks, once or twice weekly. Home practice: Encouraged to do 30 min of tai chi daily, and after intervention as well.	Mean age: 50.9 (12.5) (*n* = 75) Components: Aerobic exercise: low intensity movements, and dynamic and static stretching with a gradual increase in duration of exercise. Duration and frequency: Aerobic exercise, 24 weeks, twice weekly for 60 min. Home practice: Encouraged to do 30 min of aerobic exercise daily, and after intervention as well.	226 adults with fibromyalgia, 21 years or older, who met ACR 1990 and 2010 criteria, passed a mini-mental state examination, and had no other complementary and alternative medicine within the past 6 months or other serious health condition	Baseline, 24 weeks, 52 weeks	Pain improved more at 24th week than 12th week. At 24 weeks, combined tai chi groups reported significantly improved pain compared to the aerobic exercise group.	Anxiety improvement in tai chi group. Both groups showed reduced use of pain and depression medications over time.	12 week evaluation: 183 (81%) completed it 24 weeks: 181 (80%) 52 weeks: 158 (70%)	Tai Chi: 62% Aerobic exercise: 40%	154 Adverse events: Tai Chi: 117 Aerobic exercise: 37
Cash et al. (63) (Brazil)	Intervention: 8 week MBSR Population: Physician diagnosed FM female patients Mean age: Not reported (*n* = 51) Components: MBSR: Attention-focusing, body awareness, sitting meditation and multiple simple yoga postures to encourage relaxation. Duration and frequency: 8-week MBSR program; weekly 2.5-h sessions including meditation, yoga postures, and discussion. Home practice: Encouraged to do 45 min per day at home practice.	(*n* = 40) wait-list control participants	91 female FM patients 18 years and older, with physician diagnosis of FM	Baseline, post-treatment, and 2-month follow-up	Reduced stress, sleep disturbances, fatigue, and symptom severity (75% participants) in the intervention group. Reduced CAR post-treatment was nearly significant. At-home practice associated with less symptom severity.	Reductions in pain and symptom severity.	Illness-based absences: 25%	Not reported	Not reported
Davis and Zautra (66) (United States)	Intervention: 6-week MSER Population: Physician diagnosed female FM patients Mean age: 46.14 years (*n* = 39) Components: MSER: mindfulness meditation (for emotional regulation, and pain acceptance), and mindful awareness skills to build social bonds Duration and frequency: A 12-module (15 min each) online intervention for 6 weeks. Home practice: Encouraged to practice skills learned in the module over next several days. Audio recording of mindful meditation provided.	Mean age = 46.14 years (*N* = 40) Components: Health tips (HT) involved daily habits of healthy living. modules covered a health behavior concern (e.g., diet, exercise, and sleep). Duration and frequency: Same as MSER. Home practice: None	79 FM patients 18 or older, understand English, have a physician diagnosis of FM and access to the internet	Baseline, post-treatment	MSER group showed improved pain coping efficacy, positive affect, and social engagement, whereas HT either did not improve or remained unchanged. Patients with previous depression showed improved loneliness, family stress, and positive affect in the MSER but not in the HT group.	Not reported	HT: 5% MSER: 15%	HT: 63% MSER: 49%	Not reported

This table shows the trials that evaluate mind-body therapies for fibromyalgia. MBCT, mindfulness-based cognitive therapy; MBSR, mindfulness-based stress reduction; CBT, cognitive behavioral therapy; MSER, mindful socioemotional regulation intervention; RAP, recurrent abdominal pain; HRQoL, health related quality of life; FIQ, fibromyalgia impact questionnaire; VAS, visual analog scales; FIQR, fibromyalgia impact questionnaire revised; PGIC, the patient global impression of change; SF-36, short form-36; PHQ-15, patient health questionnaire; IBS-SSS, IBS symptom severity scale; STAI, the state-trait anxiety inventory; HADS, hospital anxiety and depression scale; S, Cohen perceives stress scale; PSQ, perceived stress questionnaire; BAQ, body awareness questionnaire; TAU, treatment as usual; IBS–SSS, irritable bowel syndrome symptom severity score; WSAS, work and social adjustment scale; PCS, pain catastrophizing scale; C-SOSI, symptoms of stress; BPI, brief pain inventory; S1, primary somatosensory cortex.

**TABLE 2:** Mind-body randomized control trials for IBS.

Study	Intervention and population	Comparison	Inclusion criteria	Assessments	Results (primary outcomes)	Results (secondary outcomes)	Attrition	Compliance	Adverse events
Kuttner et al. (89) (Canada)	Intervention: 4 weeks yoga Population: Adolescents with IBS, diagnosed by Rome I Mean age = 14.36 ± 2.10 (*n* = 14) Components: Yoga: focused abdominal breathing, selected poses and regulated deep relaxed breathing Duration and frequency: 1 h instructional session once followed by 4 weeks of daily practice. Home Practice: at-home practice using a DVD for 4 weeks.	Mean age = 13.83 ± 1.89 (*n* = 11) wait-list control group	28 Adolescents (11–18), Rome I	Baseline, 4 weeks	Significant reduction in gastrointestinal symptoms in yoga group.	Lower levels of functional disability, emotion-focused coping and anxiety than adolescents in the control group. Qualitative findings: Participants found Yoga helpful, enjoyable, and easy to do.	Not reported	Not reported	Not reported
Evans et al. (88) (United States)	Intervention: 6 week Iyengar yoga Population: Adolescents with IBS, diagnosed by Rome III Mean age = 16.4 (*n* = 29) Components: Iyengar yoga: Carefully selected poses for IBS ranging from standing, reclining, and seated poses, to forward bends, back bends, and supported inversions. Duration and frequency: 6 weeks of 1.5-h classes, twice per week. Home practice: Suggested but not mandatory.	Mean age not reported. wait-list control group (*n* = 22)	18 adolescents and young adults (14–26), RAP, or ROME III, English-speaking and able to provide consent	Baseline, 6 weeks	Half the participants in intervention group experienced reduction in gastrointestinal symptoms, disability, sleep problems, and fatigue compared to non-responders.	Improvement in QOL, fatigue, and physical functioning.	Yoga group: Baseline: 24% (10/12 yoga classes)	Not reported	Not reported
Tavakoli et al. (87) (Iran)	Intervention: 7 week laughter yoga Population: Adults with IBS diagnosed by Rome III Mean age: 33.10 (SD 9.49) (*n* = 20) Components: Group A laughter yoga: laughter techniques, deep breathing, relaxation, informative sessions Duration and frequency: One session of laughter yoga therapy for each week for 7 weeks. Home practice: None	Mean age: 32.38 (9.23) (*n* = 20) Group B anti-anxiety medication. Sertraline (50–200 mg per day) Mean age: 31.72 (9.02) (*n* = 20) Group C Symptomatic therapy: Intervention was not the same in the symptomatic treatment group. However, no anti-anxiety medications.	60 patients (18–50), ROME III	Baseline, follow-up	Reduced symptom severity in yoga group in comparison to anti-anxiety group. Change in anxiety was not statistically significant	Not reported	Not reported	Not reported	Not reported
Schumann et al. (86) (Germany)	Intervention: 12 week Hatha yoga Population: Adults with IBS diagnosed by Rome III Mean age: Not reported (*n* = 30) Components: Hatha yoga: Mantra meditation, yoga nidra which comprises of deep relaxation techniques, and postures designed to influence digestive organs. Duration and frequency: 75 min weekly hatha yoga sessions for 12 weeks. Home Practice: 3.5 h video provided for at-home practice.	Mean age: Not reported (*n* = 29) Low-FODMAP diet Components: 4 sessions of nutritional counseling lasting 60–90 min. Low-FODMAP recipes, and other resources. Duration: 12 weeks	59 Male and female IBS patients, ROME III	Baseline, 12 weeks, 24 weeks	No significant group differences.	The yoga group demonstrated a statistically significant improvement in the physical symptoms, perceived stress, and anxiety when compared to the low-FODMAP group.	Yoga: 5.81% FODMAP: 13.8%	Yoga class: 14.9 ± 7.99/24 Yoga home practice: 96.3 ± 38.2 min FODMAP: 2.62 ± 0.68/3 sessions Diet compliance: 70.7 ± 32.0 The self-reported 100 visual analog scale 67.7 ± 2.3 on the nutritionists-reported 100 visual analog scale	Yoga: 2 reported FODMAP: 3 events with 1 serious.
Taneja et al. (90) (India)	Intervention: 8 week yoga Population: Male IBS patients diagnosed by Rome II Mean age (both groups) = 30.9 ± 6.79 (*n* = 9) Components: Yoga: Surya nadi pranayama (right-nostril breathing), and 12 asanas Duration and frequency: twice a day for 2 months Home practice: Not reported	Mean age (both groups) = 30.9 ± 6.79 (*n* = 12) Conventional group: loperamide Duration and Frequency: 2–6 mg/day for 2 months	22 male IBS patients diagnosed through Rome II criteria. IBS-D only.	Baseline, 1 and 2 months	Yoga showed greater improvement in autonomic symptom score, and bowel symptom score in contrast to conventional group. Increased parasympathetic reactivity was found at the end of 2 months in yogic group.	In comparison to conventional group, significant difference in reduction of anxiety was discovered in yoga group at 1 month.	Yoga: 5% Conventional treatment: 0%	Not reported	Not reported
Kavuri et al.(91) (United States)	Intervention: 12 week Remedial Yoga Population: Adult IBS patients diagnosed by Rome III Mean age: 45.8 ± 12.7 (*n* = 25) Components: Remedial yoga module: Breathing practices, instant and quick relaxation exercises, variety of postures, regulated breathing, meditation, and closing affirmation. Also encouraged to voluntarily reduce their medicine intake to 3 times per week. Duration and frequency: One hour three times per week for 12 weeks Home practice: Not reported	Mean age: age = 41.2 ± 12.8 (*n* = 26) Combination group: Yoga intervention and conventional treatment. Mean age: 45.8 ± 12.9 (*n* = 27) Wait-list control Group: Conventional treatment continued, and encouraged to walk 1 h three times a week.	Individuals aged 18 and above, ROME III criteria	Baseline, 6 and 12 weeks	In comparison to wait-list, there were significant improvements in symptom severity and QOL in yoga and combination groups	Significant improvement in anxiety, gastrointestinal specific anxiety, physical flexibility, and autonomic functions in both yoga and combination groups yielded less frequent use of medications. Self-reported findings from 80% of participants indicate better sleep, and energy levels associated with yoga.	Yoga: 24% Combination group: 21%	Yoga: 90% Combination group: 90%	Three participants (yoga = 2; combination = 1) complained of lower back pain which was relieved and participants completed the program. 1 deceased due to cardiac event in wait-list group. No adverse events related to the intervention
Shahabi et al. (92) (United States)	Intervention: 16 biweekly Iyengar yoga Population: Adult IBS patients diagnosed by Rome III Mean age = 34.7 ± 11.6 (*n* = 17) Components: Iyengar yoga postures consisting of seated poses, inversions, backbends, twists, and supine poses. Duration and frequency: Iyengar Yoga 16 biweekly group sessions for 60 min and Home practice: Encouraged	Mean age = 39 ± 15.0 (*n* = 10) Moderate intensity outdoor walking, non-aerobic led by an instructor. Additional discussion during each walking session Duration and frequency: Same as yoga Home practice: Encouraged	35 IBS patients (18–65) ROME III criteria, Male and Female	Baseline, 8 weeks, 6 months	Yoga group shows decreases in symptom severity, visceral sensitivity, whereas walking group shows decreases in symptom severity and anxiety. However, GI symptoms returned to baseline levels at 6-month follow-up for yoga group, whereas for walking they continued to decrease. Home practice was common in walking group.	Walking group shows improvements in negative affects, and state anxiety.	Yoga: 14.3% Walking: 8.6%	Yoga: 14.2 ± 2.0/16 classes Walking: 13.8 ± 3.1/16 classes	Not reported
Everitt et al. (77) (United Kingdom)	Intervention: 8 week Telephone-CBT, web-CBT Population: Adult patients with refractory IBS diagnosed by Rome III Mean age: not reported Therapist-delivered telephone CBT (telephone-CBT group) (*n* = 119) Duration and frequency: 1-h telephone sessions, 8 times Web-based CBT with minimal therapist support (web-CBT group) (*n* = 99) Duration and frequency: 2.5-h therapist support via phone Home practice: Not applicable	(*n* = 105) Treatment as usual (TAU group): continuation of current medication, and consultant follow ups	558 adults with refractory IBS. Rome III IBS-SSS ≥ 7, offered first-line therapies and IBS symptoms for 12 months or longer.	3 months, 6 months, 12 months, 24-month	Sustained improvements in both CBT groups (telephone CBT and web CBT) at 24 months. Symptom severity was lower in the telephone-CBT group.	Lower anxiety in the telephone-CBT group	Not reported	Telephone CBT: 29 (16%) Web CBT: 57 (31%) TAU: 0 (0%)	41 adverse events; gastrointestinal, musculoskeletal, and psychological
Henrich et al. (68) (United Kingdom)	Intervention: 6 week MBCT Population: Adult female IBS patients diagnosed by Rome III Mean age: 35.58 (*n* = 36) Components: MBCT-IBS: Body awareness, behaviors and emotional reactivity, and coping; session included meditation, psychoeducation relevant to IBS, discussion of home practice, and inquiry. Duration and frequency: 2 h sessions per week for 6 weeks. A 1-week break after the fifth session. Home practice: 1 h	Mean age: 35.48 (*n* = 31) waitlist control condition	67 female patients with IBS (aged 18–65 years), Rome III, English fluency, normal vision	Baseline, after 2 treatment sessions, at posttreatment, and at 6-week follow-up.	Greater reductions in IBS symptoms in MBCT than waitlist and improvements in quality of life maintained post-treatment in MBCT group.	Improvement in pain levels and pain catastrophizing at posttreatment.	34% (*n* = 23) of participants withdrew from the study	56 participants completed the intratreatment assessment, 48 for the posttreatment assessment and 44 for the follow-up assessment	Not reported
Zernicke et al. (64) (Canada)	Intervention: 8 week MBSR Population: Adults with IBS diagnosed by Rome III Mean age: 45 (12.4) (*n* = 43) Components: MBSR: Participants were taught meditation techniques, body awareness skills, general psychoeducation, and Yoga in a didactic classroom format. Duration and frequency: 8-week MBSR program group sessions of 90 min duration plus a 3-h morning workshop between weeks 6 and 7. Home practice: Encouraged. 52-page booklet and two CDs to aid home practice.	Mean age: 44 (SD012.6) (*n* = 47) wait-list control participants	90 patients diagnosed with IBS (18 or older), Rome III criteria diagnosis by gastroenterologist	Pre- and post-intervention and at 6-month follow-up	Reduced symptom severity for both groups (waitlist and MBSR), with more improvement in MBSR group (50% participants and 30.7% reduction) than wait-list (21% participants and 5.2% reduction)	Both improved in overall mood, QOL, and spirituality and maintained at 6 months. Stress symptoms reduced from pre- to post-intervention for the MBSR treatment group, with results maintained at 6-month follow-up	MBSR: 44% Waitlist: 23% 6 month follow up MBSR: 17% Waitlist: 6%	The mean number of MBSR classes attended: six out of nine (including 3-h silent retreat).	Not reported
Ljótsson et al. (65) (Sweden)	Intervention: 10 week CBT Population: IBS patients diagnosed by Rome III Mean age: 36.4 (10.1) (*N* = 42) Components: CBT-protocol: Self-awareness, and mindfulness exercises in the form of a text based self-help manual, divided into five steps of treatment. Therapist support was provided and participants were encouraged to send one message per week. Duration and frequency: 10 weeks Home practice: Encouraged to practice daily	Mean age: 32.8 (8.6) (*N* = 43) Wait-list control Online discussion forum with general discussion regarding IBS.	85 self-referred IBS-patients recruited between May and July 2008. Diagnosis of IBS given by a physician and Rome III criteria for IBS; telephone interviews conducted for selected participants to reaffirm this.	Pre-treatment, post-treatment, and 3 month follow-up	CBT treatment group reported a 42% decrease in primary symptoms, whereas control group reports a 12% increase.	Treatment group improved on all secondary outcome measures: QOL, GI-specific anxiety, depression and general functioning	Four participants did not complete post-treatment assessment in treatment group.	Twenty-nine (74%) of the 42 participants in treatment group finished 5th step of treatment. All participants in the control group finished the posttreatment assessment	Not reported
Lackner et al. (78, 79) (United States)	Intervention: 10 week CBT (standard vs. minimal contact) Population: Adult IBS patients diagnosed by Rome II Mean age rapid responders (RRs): 47.3 (17.7) (*N* = 21) Mean age Non-rapid responders (NRRs) 46.0 (16.2) (*N* = 50) Components: CBT: Self-regulation, self-awareness, negative thoughts, and coping Duration and frequency: Standard CBT [S-CBT]: 10 weekly 1-h sessions of CBT Minimal contact CBT [MC-CBT]: Four 1-h sessions over 10 weeks. Primary reliance on self-study material. Two 10 min phone calls at week 3 and 7 for troubleshooting. Home practice: Weekly homework assigned	Mean age: 49.7 (17.6) (*N* = 23) Wait- list control	71 individuals, aged 18–70 years, IBS symptoms and fulfill Rome II criteria (moderate severity at least) without other GI comorbidities	Baseline, 2 weeks after treatment, 3-month follow up	Both CBT versions (minimal contact and standard interventions) were significantly better than control. RRs identified as participants with decrease in severity scores of 50 or greater by week 4. 30% of CBT participants were RRs. 95% of the RRs maintained their scores after the intervention and at 3-month follow-up despite having more severe IBS scores at baseline.	Improved quality of life and IBS symptom severity in comparison to control condition, however not in psychological improvement.	21% (16 participants) dropped out.	Follow-up data missing for 16% of participants	Not reported

This table shows the trials that evaluate mind-body therapies for Irritable Bowel Syndrome. MBCT, mindfulness-based cognitive therapy; MBSR, mindfulness-based stress reduction; CBT, cognitive behavioral therapy; MSER, mindful socioemotional regulation intervention; RAP, recurrent abdominal pain; HRQoL, health related quality of life; FIQ, fibromyalgia impact questionnaire; VAS, visual analog scales; FIQR, fibromyalgia impact questionnaire revised; PGIC, the patient global impression of change; SF-36, short form-36; PHQ-15, patient health questionnaire; IBS-SSS, IBS symptom severity scale; STAI, the state-trait anxiety inventory; HADS, hospital anxiety and depression scale; CPSS, Cohen perceives stress scale; PSQ, perceived stress questionnaire; BAQ, body awareness questionnaire; TAU, treatment as usual; IBS–SSS, irritable bowel syndrome symptom severity score; WSAS, work and social adjustment scale; PCS, pain catastrophizing scale; C-SOSI, symptoms of stress; BPI, brief pain inventory; RAP, recurrent abdominal pain.

### 6.1 Mindfulness

Mindfulness is used to treat both IBS and FM (20, 58–66). A recent meta-analysis suggests mindfulness and acceptance-based interventions result in moderate improvements in pain, sleep, quality of life, anxiety and depression in FM (67). In IBS, a recent systematic review highlights improvements in psychological wellbeing, catastrophizing, and pain coping efficacy with mindfulness (20). Another online mindfulness trial demonstrated a significant improvement in the IBS quality of life and GI-Specific Anxiety, with 42% of intervention participants reporting decreased IBS symptoms compared to a 12% increase in controls (65). Other trials show significant improvements in IBS symptom severity, quality of life and anxiety with mindfulness therapy, compared to controls (64, 65, 68) (Table 2). Moreover, the improvement in symptom severity was maintained at a 6 month follow-up in the intervention group (64).

In FM, a web-based mindful socioemotional regulation intervention improved pain, stress coping, social engagement, and loneliness in comparison to a health education control group (20, 66) (Table 1). Another randomized controlled trial found significant decreases in stress, and sleep disturbances, suggesting those with greater at-home practice had decreased symptom severity (63). The proposed mechanisms of action of mindfulness is through decreased sympathetic outflow and HPA axis activation (57), with associated changes in brain connectivity resulting in enhanced self-regulation through the modulation of emotions, self-compassion, and body awareness (69, 70).

### 6.2 Cognitive behavioral therapy

Cognitive Behavioral Therapy (CBT) involves altering unhelpful patterns of thinking (cognitive bias) to alleviate stress, and improve self-regulation (68). CBT has also shown promising outcomes with reducing catastrophizing through Acceptance and Commitment Therapy. This allows participants to reflect on their thoughts and sensations, effectively reducing psychological symptoms and facilitating pain acceptance, thus improving quality of life (71, 72). Although the mechanism behind such psychological interventions is unclear, an improvement in illness-specific thoughts, beliefs and perceptions or cognitive bias has been postulated (73). In both IBS and FM, CBT results in decreased symptom severity, improved mental health and quality of life (32, 65, 68, 74–79).

The Cognitive Activation Theory of Stress hypothesizes that insomnia causes changes in the HPA axis, the central nervous system, and increases sympathetic activity, causing higher pain sensitivity (32). In turn, pain prevents restful sleep. Patients can undergo CBT specifically aimed at treating insomnia to reduce chronic arousal, improve sleep quality, and consequently pain.

In IBS, there have been four trials of CBT which reported benefits on symptom severity (65, 68, 77–79) (Table 2). A 24-month follow up comparing telephone CBT, web CBT and a treatment as usual group found greatest reduction of symptom severity in the telephone-CBT group (77). Patients receiving a 10 week course of CBT who achieved a positive response by week 4 (termed as rapid responders) experienced symptomatic reduction that was maintained at 3 month follow up (78). Similarly, a trial of CBT in female IBS patients found reduced pain catastrophizing, and improved quality of life compared to waitlist controls (68). A meta-analysis demonstrates CBT was most effective with long-term or continuous home practice (80, 81).

### 6.3 Yoga

Yoga combines techniques of different MBIs including breath work, movement, and meditation, showing promising benefits in chronic diseases such as cancer, IBS, as well as mental illnesses (82–84). Yoga improves balance, strength, and mobility, and allows non-judgmental observation of thoughts. Schumann et al. suggest it is a safe and feasible therapy for IBS because it improves symptom severity, quality of life, physical functioning and anxiety (85) (Table 2), however, more high quality clinical trials are needed to determine efficacy (85–92). The proposed mechanisms includes changes in autonomic outflow, as well as changes in central connectivity in the brain (69, 93–95). Moreover, breathing influences autonomic activity; in yoga, this is demonstrated through decreased sympathetic and increased parasympathetic activity (3, 59). In comparison to other therapies such as Mindfulness-based stress reduction, a low-FODMAP diet, and physical exercise (but not CBT), yoga was shown to be superior in improving quality of life, GI symptom severity and reducing stress and anxiety (3). Yoga programs inclusive of different breathing exercises, postures and meditation have beneficial effects on symptom severity in comparison to CBT; thus yoga programs with multiple modalities of mindfulness may provide more benefits (3). Although larger studies are needed, preliminary studies in adults and adolescents suggest that clinically meaningful improvement in IBS symptoms and sleep quality is experienced from yoga (86–89). However, qualitative studies demonstrate the need for better adherence strategies, social support, and yoga programs tailored for IBS (88, 89). For example, yoga delivered in a group setting was found to be more effective with engaged participants (71).

Yoga also demonstrates benefits in FM (59, 96–98) (Table 1). A trial with female FM patients comparing a Yoga Awareness program to a wait-listed control showed decreased anxiety (by 42.2%), depression (41.5%), emotional distress (30%), and fatigue (29.9%) in the intervention group (31, 59, 60). Sustained improvements were seen at 3 month follow-up, with greater impact when adhering to at-home yoga practice (59, 60). A pilot study with daily home practice showed reductions in catastrophization and pain, which were maintained at 6 month follow-up (98). A gentle Hatha Yoga program improved FM physical symptoms, assessed with the Fibromyalgia Impact Questionnaire (97). Interestingly, Yoga in combination with Tui Na massage (targeting meridians and acupuncture points on the body) showed promising results in pain reduction (96). Thus, multiple modalities of yoga demonstrate clinical benefit in FM.

## 7 Limitations and future directions

A strong relationship between FM and IBS is evident through their pathogenesis. The current evidence base for MBIs in the treatment of IBS and FM is growing. Studies have demonstrated multiple physical and mental health benefits, along with safety and feasibility. To our knowledge, high quality studies such as large randomized control trials assessing the efficacy of MBIs in co-diagnosed patients with IBS and FM are lacking. Therefore, we recommend that future studies testing the feasibility and efficacy of MBIs should use an active comparator groups and be tailored toward the patient to increase intervention effectiveness. Gaps in the literature include assessment of optimal MBI duration, frequency, components (single vs. multimodal) and delivery (online vs. in-person).

Our review has several limitations. First, the heterogeneity of the MBIs chosen for discussion included only the most investigated interventions among IBS and FM patients. Second, assessing MBI efficacy is challenging given the examined studies differ greatly in their methodologies. This limits the generalizability of the results, and the specific recommendations (MBI type, dose, and frequency) that can be made for co-diagnosed IBS and FM.

Until further data from high-quality trials are available to inform a definitive approach to yoga interventions in co-diagnosed patients, yoga practice involving postures, breathing, and meditation may be recommended at a dose of 30 min daily, five times weekly. These recommendations are in parallel to widely accepted physical activity guidelines and from studies that demonstrate similar integrated approach to yoga intervention and dosage achieve improved outcomes (99, 100).

Lastly, studies should also evaluate potential mechanisms of action of MBIs such as microbiome alteration, neuroendocrine/neuroimmune responses, and autonomic outflow.

## 8 Conclusion

Negative impacts on patient quality of life and mental health arising from comorbid FM and IBS, and limited data on co-diagnosed patients warrant study of effective interventions. MBIs such as CBT and yoga are impactful and leverage one of many potential pathophysiological mechanisms. Future interventions should aim toward tailoring yoga programs in combination with other MBIs to meet the needs of IBS and FM patients.

## Author contributions

ZI drafted the manuscript. AD’S, MR, and YN critically revised the manuscript for important intellectual content. All authors have reviewed and approved the final manuscript.

## References

[1] Chey W Kurlander J Eswaran S. Irritable bowel syndrome: a clinical review. *JAMA.* (2015). 313:949–58. 10.1001/jama.2015.0954 25734736

[2] Laskaratos F Goodkin O Thoua N Murray C. Irritable bowel syndrome. *Medicine.* (2015) 43:266–70. 10.1016/j.mpmed.2015.02.010

[3] D’Silva A MacQueen G Nasser Y Taylor L Vallance J Raman M. Yoga as a therapy for irritable bowel syndrome. *Dig Dis Sci.* (2020) 65:2503–14. 10.1007/s10620-019-05989-6 31832970

[4] Warnock J Clayton A. Chronic episodic disorders in women. *Psychiatr Clin North Am.* (2003) 26:725–40. 10.1016/S0193-953X(03)00042-X 14563106

[5] Buono J Carson R Flores N. Health-related quality of life, work productivity, and indirect costs among patients with irritable bowel syndrome with diarrhea. *Health Qual Life Outcomes.* (2017) 15:1–8. 10.1186/s12955-017-0611-2 28196491 PMC5310011

[6] Erdrich S Hawrelak J Myers S Harnett J. A systematic review of the association between fibromyalgia and functional gastrointestinal disorders. *Therap Adv Gastroenterol.* (2020) 13:1756284820977402. 10.1177/1756284820977402 33343707 PMC7727037

[7] Sluka K Clauw D. Neurobiology of fibromyalgia and chronic widespread pain. *Neuroscience.* (2016) 338:114. 10.1016/j.neuroscience.2016.06.006 27291641 PMC5083139

[8] Ranum R Toussaint L Whipple M Vincent A. Predictive bidirectional relations between pain, fatigue, and dyscognition in fibromyalgia. *Mayo Clin Proc Innov Qual Outcomes.* (2022) 6:143–7. 10.1016/j.mayocpiqo.2021.12.007 35243207 PMC8866045

[9] Queiroz L. Worldwide epidemiology of fibromyalgia topical collection on fibromyalgia. *Curr Pain Headache Rep.* (2013) 17:356. 10.1007/s11916-013-0356-5 23801009

[10] Rusu C Gee M Lagacé C Parlor M. Chronic fatigue syndrome and fibromyalgia in Canada: prevalence and associations with six health status indicators. *Heal Promot Chronic Dis Prev Can.* (2015) 35:3–11. 10.24095/hpcdp.35.1.02 25811400 PMC4939456

[11] Lacasse A Bourgault P Choinière M. Fibromyalgia-related costs and loss of productivity: a substantial societal burden. *BMC Musculoskelet Disord.* (2016) 17:168. 10.1186/s12891-016-1027-6 27084363 PMC4833946

[12] Clauw D. Fibromyalgia: a clinical review. *JAMA.* (2014) 311:1547–55. 10.1001/jama.2014.3266 24737367

[13] Kleykamp B Ferguson M McNicol E Bixho I Arnold L Edwards R The prevalence of psychiatric and chronic pain comorbidities in fibromyalgia: an acttion systematic review. *Semin Arthritis Rheum.* (2021) 51:166–74. 10.1016/j.semarthrit.2020.10.006 33383293

[14] Carbone F Van Den Houte K Besard L Tack C Arts J Caenepeel P Diet or medication in primary care patients with IBS: the DOMINO study–a randomised trial supported by the Belgian health care knowledge centre (KCE trials programme) and the Rome foundation research institute. *Gut.* (2022) 71:2226–32. 10.1136/gutjnl-2021-325821 35483886 PMC9554021

[15] Giorgi V Sirotti S Romano M Marotto D Ablin J Salaffi F Fibromyalgia: one year in review 2022. *Clin Exp Rheumatol.* (2022) 40:1065–72. 10.55563/clinexprheumatol/if9gk2 35748720

[16] Paine P. Review article: current and future treatment approaches for pain in IBS. *Aliment Pharmacol Ther.* (2021) 54(Suppl. 1):S75–88. 10.1111/apt.16550 34927753

[17] Pioro-Boisset M Esdaile J Fitzcharles M. Alternative medicine use in fibromyalgia syndrome. *Arthritis Care Res.* (1996) 9:13–7. 10.1002/art.1790090105 8945108

[18] Kong S Hurlstone D Pocock C Walkington L Farquharson N Bramble M The incidence of self-prescribed oral complementary and alternative medicine use by patients with gastrointestinal diseases. *J Clin Gastroenterol.* (2005) 39:138–41. 10.1097/01.mcg.0000177234.36640.68 15681910

[19] Magge S Wolf J. Complementary and alternative medicine and mind-body therapies for treatment of irritable bowel syndrome in women. *Womens Health (Lond).* (2013) 9:557–67. 10.2217/WHE.13.57 24161308

[20] Toivonen K Zernicke K Carlson L. Web-based mindfulness interventions for people with physical health conditions: systematic review. *J Med Internet Res.* (2017) 19:1–35. 10.2196/jmir.7487 28860106 PMC5599726

[21] Bulzacka E Lavault S Pelissolo A Bagnis Isnard C. Mindful neuropsychology: mindfulness-based cognitive remediation. *Encephale.* (2018) 44:75–82. 10.1016/j.encep.2017.03.006 28483271

[22] McClintock A McCarrick S Garland E Zeidan F Zgierska A. Brief mindfulness-based interventions for acute and chronic pain: a systematic review. *J Alternat Complement Med.* (2019) 25:265–78. 10.1089/acm.2018.0351 30523705 PMC6437625

[23] Wolfe F Clauw D Fitzcharles M Goldenberg D Häuser W Katz R 2016 Revisions to the 2010/2011 fibromyalgia diagnostic criteria. *Semin Arthritis Rheum.* (2016) 46:319–29. 10.1016/j.semarthrit.2016.08.012 27916278

[24] Whitehead W Palsson O Jones K. Systematic review of the comorbidity of irritable bowel syndrome with other disorders: what are the causes and implications? *Gastroenterology.* (2002) 122:1140–56. 10.1053/gast.2002.32392 11910364

[25] Heidari F Afshari M Moosazadeh M. Prevalence of fibromyalgia in general population and patients, a systematic review and meta-analysis. *Rheumatol Int.* (2017) 37:1527–39. 10.1007/s00296-017-3725-2 28447207

[26] Van Oudenhove L Levy R Crowell M Drossman D Halpert A Keefer L Biopsychosocial aspects of functional gastrointestinal disorders: how central and environmental processes contribute to the development and expression of functional gastrointestinal disorders. *Gastroenterology.* (2016) 150:1355–1367.e2. 10.1053/j.gastro.2016.02.027 27144624 PMC8809487

[27] Hu Z Li M Yao L Wang Y Wang E Yuan J The level and prevalence of depression and anxiety among patients with different subtypes of irritable bowel syndrome: a network meta-analysis. *BMC Gastroenterol.* (2021) 21:23. 10.1186/s12876-020-01593-5 33413140 PMC7791666

[28] Zamani M Alizadeh-Tabari S Zamani V. Systematic review with meta-analysis: the prevalence of anxiety and depression in patients with irritable bowel syndrome. *Aliment Pharmacol Ther.* (2019) 50:132–43. 10.1111/apt.15325 31157418

[29] Miller V Hopkins L Whorwell P. Suicidal ideation in patients with irritable bowel syndrome. *Clin Gastroenterol Hepatol.* (2004) 2:1064–8. 10.1016/S1542-3565(04)00545-2 15625650

[30] Duque L Fricchione G. Fibromyalgia and its new lessons for neuropsychiatry. *Med Sci Monit Basic Res.* (2019) 25:169–78. 10.12659/MSMBR.915962 31273184 PMC6636402

[31] Bravo C Skjaerven L Guitard Sein-Echaluce L Catalan-Matamoros D. Effectiveness of movement and body awareness therapies in patients with fibromyalgia: a systematic review and meta-analysis. *Eur J Phys Rehabil Med.* (2019) 55:646–57. 10.23736/S1973-9087.19.05291-2 31106558

[32] McCrae C Curtis A Craggs J Deroche C Sahota P Siva C Protocol for the impact of CBT for insomnia on pain symptoms and central sensitisation in fibromyalgia: a randomised controlled trial. *BMJ Open.* (2020) 10:e033760. 10.1136/bmjopen-2019-033760 32933953 PMC7493102

[33] Staud R Robinson M Price D. temporal summation of second pain and its maintenance are useful for characterizing widespread central sensitization of fibromyalgia patients. *J Pain.* (2007) 8:893–901. 10.1016/j.jpain.2007.06.006 17681887 PMC2174917

[34] Chang L Mayer E Munakata J Silverman D Berman S Gilmore S Differences in left prefrontal activation to visceral and somatic stimuli assessed by O-15-water PET in female patients with irritable bowel syndrome (IBS) and fibromyalgia. *Gastroenterology.* (1998) 114:A732. 10.1016/S0016-5085(98)83000-X

[35] Vanner S Greenwood-Van Meerveld B Mawe G Shea-Donohue T Verdu E Wood J Fundamentals of neurogastroenterology: basic science. *Gastroenterology.* (2016) 150:1280–91. 10.1053/j.gastro.2016.02.018 27144618 PMC5673591

[36] Trucharte A Leon L Castillo-Parra G Magán I Freites D Redondo M. Emotional regulation processes: influence on pain and disability in fibromyalgia patients. *Clin Exp Rheumatol.* (2020) 38:40–6. 31928594

[37] Ichesco E Puiu T Hampson J Kairys A Clauw D Harte S Altered fMRI resting-state connectivity in individuals with fibromyalgia on acute pain stimulation. *Eur J Pain.* (2016) 20:1079–89. 10.1002/ejp.832 26773435

[38] Gracely R Petzke F Wolf J Clauw D. Functional magnetic resonance imaging evidence of augmented pain processing in fibromyalgia. *Arthritis Rheum.* (2002) 46:1333–43. 10.1002/art.10225 12115241

[39] Mertz H Morgan V Tanner G Pickens D Price R Shyr Y Regional cerebral activation in irritable bowel syndrome and control subjects with painful and nonpainful rectal distention. *Gastroenterology.* (2000) 118:842–8. 10.1016/S0016-5085(00)70170-3 10784583

[40] Hong J Kilpatrick L Labus J Gupta A Jiang Z Ashe-McNalley C Patients with chronic visceral pain show sex-related alterations in intrinsic oscillations of the resting brain. *J Neurosci.* (2013) 33:11994–2002. 10.1523/JNEUROSCI.5733-12.2013 23864686 PMC3713732

[41] Chang L Mayer E Johnson T Fitzgerald L Naliboff B. Differences in somatic perception in female patients with irritable bowel syndrome with and without fibromyalgia. *Pain.* (2000) 84:297–307. 10.1016/S0304-3959(99)00215-8 10666535

[42] Mazur M Furgała A Jabłoński K Mach T Thor P. Autonomic nervous system activity in constipation-predominant irritable bowel syndrome patients. *Med Sci Monit.* (2012) 18:CR493. 10.12659/MSM.883269 22847198 PMC3560712

[43] Chang L. The role of stress on physiologic responses and clinical symptoms in irritable bowel syndrome. *Gastroenterology.* (2011) 140:761–5. 10.1053/j.gastro.2011.01.032 21256129 PMC3039211

[44] Albayrak B Süsgün S Küçükakkaş O Akbaş F Yabaci A Özçelik S. Investigating of relation between fibromyalgia syndrome and intestinal microbiota. *Mikrobiyol Bull.* (2021) 55:146–60. 10.5578/mb.20219903 33882648

[45] Duan R Zhu S Wang B Duan L. Alterations of gut microbiota in patients with irritable bowel syndrome based on 16s rRNA-targeted sequencing: a systematic review. *Clin Transl Gastroenterol.* (2019) 10:e00012. 10.14309/ctg.0000000000000012 30829919 PMC6407812

[46] Magne F Gotteland M Gauthier L Zazueta A Pesoa S Navarrete P The firmicutes/bacteroidetes ratio: a relevant marker of gut dysbiosis in obese patients? *Nutrients.* (2020) 12:1474. 10.3390/nu12051474 32438689 PMC7285218

[47] Huang Y Shi X Li Z Shen Y Shi X Wang L Possible association of firmicutes in the gut microbiota of patients with major depressive disorder. *Neuropsychiatr Dis Treat.* (2018) 14:3329–37. 10.2147/NDT.S188340 30584306 PMC6284853

[48] Riedl A Schmidtmann M Stengel A Goebel M Wisser A Klapp B Somatic comorbidities of irritable bowel syndrome: a systematic analysis. *J Psychosom Res.* (2008) 64:573–82. 10.1016/j.jpsychores.2008.02.021 18501257

[49] Yavne Y Amital D Watad A Tiosano S Amital H. A systematic review of precipitating physical and psychological traumatic events in the development of fibromyalgia. *Semin Arthritis Rheum.* (2018) 48:121–33. 10.1016/j.semarthrit.2017.12.011 29428291

[50] Henningsen P Zimmermann T Sattel H. Medically unexplained physical symptoms, anxiety, and depression: a meta-analytic review. *Psychosom Med.* (2003) 65:528–33. 10.1097/01.PSY.0000075977.90337.E7 12883101

[51] Vandvik P Wilhelmsen I Ihlebæk C Farup P. Comorbidity of irritable bowel syndrome in general practice: a striking feature with clinical implications. *Aliment Pharmacol Ther.* (2004) 20:1195–203. 10.1111/j.1365-2036.2004.02250.x 15569123

[52] Choy E. The role of sleep in pain and fibromyalgia. *Nat Rev Rheumatol.* (2015) 11:513–20. 10.1038/nrrheum.2015.56 25907704

[53] Andrade A Vilarino G Sieczkowska S Coimbra D Bevilacqua G Steffens R. The relationship between sleep quality and fibromyalgia symptoms. *J Health Psychol.* (2020) 25:1176–86. 10.1177/1359105317751615 29310453

[54] Bigatti S Hernandez A Cronan T Rand K. Sleep disturbances in fibromyalgia syndrome: relationship to pain and depression. *Arthritis Care Res.* (2008) 59:961–7. 10.1002/art.23828 18576297 PMC3691959

[55] Vincent A Benzo R Whipple M McAllister S Erwin P Saligan L. Beyond pain in fibromyalgia: insights into the symptom of fatigue. *Arthritis Res Ther.* (2013) 15:221. 10.1186/ar4395 24289848 PMC3978642

[56] Reynolds K Vernon S Bouchery E Reeves W. The economic impact of chronic fatigue syndrome. *Cost Eff Resour Alloc.* (2004) 2:4. 10.1186/1478-7547-2-4 15210053 PMC449736

[57] Wahbeh H Elsas S Oken B. Mind–body interventions: applications in neurology. *Neurology.* (2008) 70:2321. 10.1212/01.wnl.0000314667.16386.5e PMC2882072 18541886

[58] Shorey S Demutska A Chan V Siah K. Adults living with irritable bowel syndrome (IBS): a qualitative systematic review. *J Psychosom Res.* (2021) 140:110289. 10.1016/j.jpsychores.2020.110289 33227554

[59] Carson J Carson K Jones K Bennett R Wright C Mist SD. A pilot randomized controlled trial of the yoga of awareness program in the management of fibromyalgia. *Pain.* (2010) 151:530–9. 10.1016/j.pain.2010.08.020 20946990 PMC5568071

[60] Carson J Carson K Jones K Mist S Bennett R. Follow-up of yoga of awareness for fibromyalgia: results at 3 months and replication in the wait-list group. *Clin J Pain.* (2012) 28:804–13. 10.1097/AJP.0b013e31824549b5 22751025 PMC5568073

[61] Schmidt S Grossman P Schwarzer B Jena S Naumann J Walach H. Treating fibromyalgia with mindfulness-based stress reduction: results from a 3-armed randomized controlled trial. *Pain.* (2011) 152:361–9. 10.1016/j.pain.2010.10.043 21146930

[62] Wang C Schmid C Fielding R Harvey W Reid K Price L Effect of tai chi versus aerobic exercise for fibromyalgia: comparative effectiveness randomized controlled trial. *BMJ.* (2018) 360:1–14. 10.1136/bmj.k851 29563100 PMC5861462

[63] Cash E Ph D Salmon P Ph D Weissbecker I Ph D Mindfulness meditation alleviates fibromyalgia symptoms in women: results of a randomized clinical trial. *Ann Behav Med.* (2015) 49:319–30. 10.1007/s12160-014-9665-0 25425224 PMC4802162

[64] Zernicke K Campbell T Blustein P Fung T Johnson J Bacon S Mindfulness-based stress reduction for the treatment of irritable bowel syndrome symptoms: a randomized wait-list controlled trial. *Int J Behav Med.* (2013) 20:385–96. 10.1007/s12529-012-9241-6 22618308

[65] Ljótsson B Falk L Vesterlund A Hedman E Lindfors P Rück C Internet-delivered exposure and mindfulness based therapy for irritable bowel syndrome–a randomized controlled trial. *Behav Res Ther.* (2010) 48:531–9. 10.1016/j.brat.2010.03.003 20362976

[66] Davis M Zautra A. An online mindfulness intervention targeting socioemotional regulation in fibromyalgia: results of a randomized controlled trial. *Ann Behav Med.* (2013) 46:273–84. 10.1007/s12160-013-9513-7 23670111

[67] Haugmark T Hagen K Smedslund G Zangi H. Mindfulness- and acceptance-based interventions for patients with fibromyalgia – a systematic review and meta-analyses. *PLoS One.* (2019) 14:e0221897. 10.1371/journal.pone.0221897 31479478 PMC6719827

[68] Henrich J Gjelsvik B Surawy C Evans E Martin M. A randomized clinical trial of mindfulness-based cognitive therapy for women with irritable bowel syndrome-effects and mechanisms. *J Consult Clin Psychol.* (2020) 88:295–310. 10.1037/ccp0000483 32134291

[69] Hölzel B Lazar S Gard T Schuman-Olivier Z Vago D Ott U. How does mindfulness meditation work? Proposing mechanisms of action from a conceptual and neural perspective. *Perspect Psychol Sci.* (2011) 6:537–59. 10.1177/1745691611419671 26168376

[70] Alsubaie M Abbott R Dunn B Dickens C Keil T Henley W Mechanisms of action in mindfulness-based cognitive therapy (MBCT) and mindfulness-based stress reduction (MBSR) in people with physical and/or psychological conditions: a systematic review. *Clin Psychol Rev.* (2017) 55:74–91. 10.1016/j.cpr.2017.04.008 28501707

[71] Adler-Neal A Zeidan F. Mindfulness meditation for fibromyalgia: mechanistic and clinical considerations. *Curr Rheumatol Rep.* (2017) 19:59. 10.1007/s11926-017-0686-0 28752493 PMC5693231

[72] Galvez-Sánchez C Montoro C Moreno-Padilla M Reyes Del Paso G De La Coba P Merwin R Clinical medicine effectiveness of acceptance and commitment therapy in central pain sensitization syndromes: a systematic review. *J Clin Med.* (2021) 10:2706. 10.3390/jcm10122706 34205244 PMC8235706

[73] Windgassen S Moss-Morris R Chilcot J Sibelli A Goldsmith K Chalder T. The journey between brain and gut: a systematic review of psychological mechanisms of treatment effect in irritable bowel syndrome. *Br J Health Psychol.* (2017) 22:701–36. 10.1111/bjhp.12250 28573818

[74] Hernando-Garijo I Jiménez-Del-Barrio S Mingo-Gómez T Medrano-De-La-Fuente R Ceballos-Laita L. Effectiveness of non-pharmacological conservative therapies in adults with fibromyalgia: a systematic review of high-quality clinical trials. *J Back Musculoskelet Rehabil.* (2022) 35:3–20. 10.3233/BMR-200282 34180405

[75] Samami E Shahhosseini Z Elyasi F. The effect of psychological interventions on the quality of life in women with fibromyalgia: a systematic review. *J Clin Psychol Med Settings.* (2021) 28:503–17. 10.1007/s10880-021-09794-0 34216335

[76] Lazaridou A Kim J Cahalan C Loggia M Franceschelli O Berna C Effects of cognitive-behavioral therapy (CBT) on brain connectivity supporting catastrophizing in fibromyalgia. *Clin J Pain.* (2017) 33:215–21. 10.1097/AJP.0000000000000422 27518491 PMC5296218

[77] Everitt H Landau S O’Reilly G Sibelli A Hughes S Windgassen S Cognitive behavioural therapy for irritable bowel syndrome: 24-month follow-up of participants in the ACTIB randomised trial. *Lancet Gastroenterol Hepatol.* (2019) 4:863–72. 10.1016/S2468-1253(19)30243-2 31492643 PMC7026694

[78] Lackner J Gudleski G Keefer L Krasner S Powell C Katz L. Rapid response to cognitive behavior therapy predicts treatment outcome in patients with irritable bowel syndrome. *Clin Gastroenterol Hepatol.* (2010) 8:426–32. 10.1016/j.cgh.2010.02.007 20170751 PMC3144205

[79] Lackner J Jaccard J Krasner S Katz L Gudleski G Holroyd K. Self administered cognitive behavior therapy for moderate to severe IBS: clinical efficacy, tolerability, feasibility. *Clin Gastroenterol Hepatol.* (2008) 6:899. 10.1016/j.cgh.2008.03.004 18524691 PMC2630498

[80] Black C Thakur E Houghton L Quigley E Moayyedi P Ford A. Efficacy of psychological therapies for irritable bowel syndrome: systematic review and network meta-analysis. *Gut.* (2020) 69:1441–51. 10.1136/gutjnl-2020-321191 32276950

[81] Radziwon C Quigley B Vargovich A Krasner S Gudleski G Mason S Do I really have to do my homework? The role of homework compliance in cognitive behavioral therapy for irritable bowel syndrome. *Behav Res Ther.* (2022) 152:104063. 10.1016/j.brat.2022.104063 35248876

[82] Glynn B Khoo E MacLeay H Duong A Cantave R Poulin P. Exploring cancer patients’ experiences of an online mindfulness-based program: a qualitative investigation. *Mindfulness (NY).* (2020) 11:1666–77. 10.1007/s12671-020-01380-z 32670431 PMC7346987

[83] Jindani F Khalsa GFS. A yoga intervention program for patients suffering from symptoms of posttraumatic stress disorder: a qualitative descriptive study. *J Altern Complement Med.* (2015) 21:401–8. 10.1089/acm.2014.0262 26133204

[84] Ewais T Begun J Kenny M Headey A Kisely S. Mindfulness-based cognitive therapy experiences in youth with inflammatory bowel disease and depression: protocol for a mixed methods qualitative study. *JMIR Res Protoc.* (2019) 8:e14432. 10.2196/14432 31342900 PMC6685121

[85] Schumann D Anheyer D Lauche R Dobos G Langhorst J Cramer H. Effect of yoga in the therapy of irritable bowel syndrome: a systematic review. *Clin Gastroenterol Hepatol.* (2016) 14:1720–31. 10.1016/j.cgh.2016.04.026 27112106

[86] Schumann D Langhorst J Dobos G Cramer H. Randomised clinical trial: yoga vs a low-FODMAP diet in patients with irritable bowel syndrome. *Aliment Pharmacol Ther.* (2018) 47:203–11. 10.1111/apt.14400 29076171

[87] Tavakoli T Davoodi N Jafar Tabatabaee T Rostami Z Mollaei H Salmani F Comparison of laughter yoga and anti-anxiety medication on anxiety and gastrointestinal symptoms of patients with irritable bowel syndrome. *Middle East J Dig Dis.* (2019) 11:212–8. 10.15171/mejdd.2019.151 31824624 PMC6895849

[88] Evans S Seidman L Lung K Sternlieb B Zeltzer L. Yoga for teens with irritable bowel syndrome: results from a mixed-methods pilot study. *Holist Nurs Pract.* (2018) 32:253–60. 10.1097/HNP.0000000000000288 30113959 PMC6283406

[89] Kuttner L Chambers C Hardial J Israel D Jacobson K Evans K. A randomized trial of yoga for adolescents with irritable bowel syndrome. *Pain Res Manag.* (2006) 11:217–23. 10.1155/2006/731628 17149454 PMC2673138

[90] Taneja I Deepak K Poojary G Acharya I Pandey R Sharma M. Yogic versus conventional treatment in diarrhea-predominant irritable bowel syndrome: a randomized control study. *Appl Psychophysiol Biofeedback.* (2004) 29:19–33. 10.1023/B:APBI.0000017861.60439.95 15077462

[91] Kavuri V Raghuram N Malamud A Selvan S. Irritable bowel syndrome: yoga as remedial therapy. *Evid Based Complement Alternat Med.* (2015) 2015:398156. 10.1155/2015/398156 26064164 PMC4438173

[92] Shahabi L Naliboff B Shapiro D. Self-regulation evaluation of therapeutic yoga and walking for patients with irritable bowel syndrome: a pilot study. *Psychol Heal Med.* (2016) 21:176–88. 10.1080/13548506.2015.1051557 26086986

[93] van Aalst J Ceccarini J Demyttenaere K Sunaert S Van Laere K. What has neuroimaging taught us on the neurobiology of yoga? A review. *Front Integr Neurosci.* (2020) 14:34. 10.3389/fnint.2020.00034 32733213 PMC7362763

[94] Naveen G Varambally S Thirthalli J Rao M Christopher R Gangadhar B. Serum cortisol and BDNF in patients with major depression-effect of yoga. *Int Rev Psychiatry.* (2016) 28:273–8. 10.1080/09540261.2016.1175419 27174729

[95] Mishra S Singh S Moheb N Khosa S Trikamji B. Changes in functional magnetic resonance imaging with yogic meditation: a pilot study. *Ayu.* (2017) 38:108. 10.4103/ayu.AYU_34_17 30254388 PMC6153914

[96] Da Silva G Lorenzi-Filho G Lage LV. Effects of yoga and the addition of Tui Na in patients with fibromyalgia. *J Altern Complement Med.* (2007) 13:1107–13. 10.1089/acm.2007.0615 18166122

[97] Rudrud L. Gentle Hatha yoga and reduction of fibromyalgia-related symptoms: a preliminary report. *Int J Yoga Therap.* (2012) 22:53–7. 10.17761/ijyt.22.1.hp278678261h5363 23070672

[98] Lazaridou A Koulouris A Dorado K Chai P Edwards R Schreiber K. The impact of a daily yoga program for women with fibromyalgia. *Int J Yoga.* (2019) 12:206–17. 10.4103/ijoy.IJOY_72_18 31543629 PMC6746047

[99] Piercy K Troiano R Ballard R Carlson S Fulton J Galuska D The physical activity guidelines for Americans. *JAMA.* (2018) 320:2020–8. 10.1001/jama.2018.14854 30418471 PMC9582631

[100] Peterson C Bauer S Chopra D Mills P Maturi R. Effects of Shambhavi Mahamudra Kriya, a multicomponent breath-based yogic practice (pranayama), on perceived stress and general well-being. *J Evid Based Complement Altern Med.* (2017) 22:788–97. 10.1177/2156587217730934 29228793 PMC5871312

